# Reviewing the efficiency of photobiomodulation therapy in oncological treatment

**DOI:** 10.3389/fonc.2024.1447653

**Published:** 2024-10-08

**Authors:** Bibhus Luitel, Tanush Duggisani, Anuj Luitel, John LaRocco

**Affiliations:** ^1^ The Ohio State University, Columbus, OH, United States; ^2^ Psychiatry and Behavioral Sciences, The Ohio State University Wexner Medical Center, Columbus, OH, United States

**Keywords:** photobiomoduation, oncology, photobiomodulation therapy, radiation therapy, low level light therapy (LLLT)

## Abstract

The aim of the present systematic review is to evaluate the effects of different photobiomodulation therapy (PBMT) approaches in oncological treatment practices. The review follows the PRISMA guidelines. Specifically, the review is composed of laser PBMT and LED PBMT. A total of 23 studies were included, 14 investigating laser PBMT and 9 examining LED PBMT. *In vitro* studies demonstrated laser PBMT’s potential to induce apoptosis and cytotoxicity in various cancer cell lines while enhancing sensitivity to chemotherapeutics and natural compounds. However, some studies highlighted divergent effects between *in vitro* (promoted proliferation) and *in vivo* xenograft models (slowed tumor growth) for certain laser wavelengths. LED PBMT studies showed blue light inhibited melanoma and pancreatic cancer cell growth, potentially via ROS generation, while red light raised concerns about enhancing oral cancer invasiveness. Both modalities mitigated treatment side effects like oral mucositis, xerostomia, peripheral neuropathy, and improved quality of life. While promising, the outcomes varied based on light parameters, cancer type, and experimental setting, necessitating further optimization of PBMT protocols through well-designed studies to establish long-term safety and efficacy across clinical scenarios.

## Introduction

Photobiomodulation therapy (PBMT) is a light therapy that utilizes non-ionizing light sources, most notably lasers and light-emitting diodes (LEDs) in the visible and near-infrared wavelength ranges (600nm-1000nm) to trigger photochemical events in cells that result in therapeutic benefits (1). It is a non-thermal process caused by photon absorption in cytochrome c oxidate, the terminal enzyme in the mitochondrial respiratory chain (2). PBMT can modulate numerous cellular processes like ATP production, reactive oxygen species generation, nitric oxide release, and transcription factor activation (1).

### Contraindications and emerging evidence

Historically, the medical community has shown initial concern about using PBMT over tumor sites due to the theoretical risk of promoting cancer cell proliferation and survival (3). However, emerging evidence suggests that PBMT may have selective benefits on healthy cells while inhibiting the growth of cancer cells. It is hypothesized that the overall effects are highly dependent on factors such as wavelength, spectrum, duration of treatment, cell types, and tumor oxygenation levels (4–6).

Perhaps an exciting emerging cancer treatment area being studied more in literature is the combination of PBMT with photosensitive pharmaceuticals, commonly called photodynamic therapy (PDT). PDT utilizes photosensitive drugs that generate cytotoxic reactive oxygen species upon photoexcitation, leading to direct tumor ablation. Interestingly, some studies have also suggested that low-level PBMT can improve the efficacy of PDT by increasing tumor oxygenation and inducing pro-oxidant states in cancer cells (7–12).

Recently, PBMT has also been used increasingly to prevent or mitigate the side effects of existing chemotherapeutics and other cancer treatments. The most notable advancements in this field have been with side effects such as oral mucositis, dermatitis, chemotherapy induced xerostomia, among others (13). This present review aims to assess recent current literature, within the last five years, to describe the clinical advancements in the use of PBMT in broad cancer treatment.

## Search methodology

### Protocol

#### Scope and eligibility criteria

The primary objective of this review is to identify and investigate experiments that discuss PBMT in oncological contexts, both *in vivo* and *in vitro* as well as clinical trials. For this review’s purpose, recent was defined as within the last five years (2019-2024). Additionally, supplemental research was conducted manually to assess emerging trends in the PBMT and low-lever irradiation practices. Lastly, only papers that were written in English were considered.

### Initial stages

Prior to implementing a search strategy, all three reviewers independently conducted a small preliminary data collection. This allowed for the review team to pilot the initial keyword parameters to qualitatively assess the relevance of the returns for queries. The preliminary data collection highlighted the need to expand certain sections, such as including keywords for different PBMT parameters like wavelength and treatment modality (laser vs LED irradiation).

### Search strategy

Searches were done using electronic queries to three major databases: Google Scholar, Scopus, and PubMed. To detect other eligible reports, the reviewers checked references from the studies that were selected. To conduct the search, the following keywords were used either alone or together:

(“Photobiomodulation” OR “Low-level light therapy” OR “Low-level laser therapy” OR “Laser phototherapy” OR “Low-intensity laser therapy” OR “light-emitting diodes”) AND (“Chemotherapy” OR “Oncology” OR “Disease free survival” OR “Tumor” or “Carcinoma”) AND “Wavelength”.

### Data management

All retrieved records were imported into reference management software, and duplicates were removed. Titles and abstracts were screened independently by two reviewers to identify potentially eligible studies. Full texts of these studies were then assessed against the eligibility criteria. Any disagreements were resolved through discussion and consensus.

## Results

### Overview

**Figure 1:**
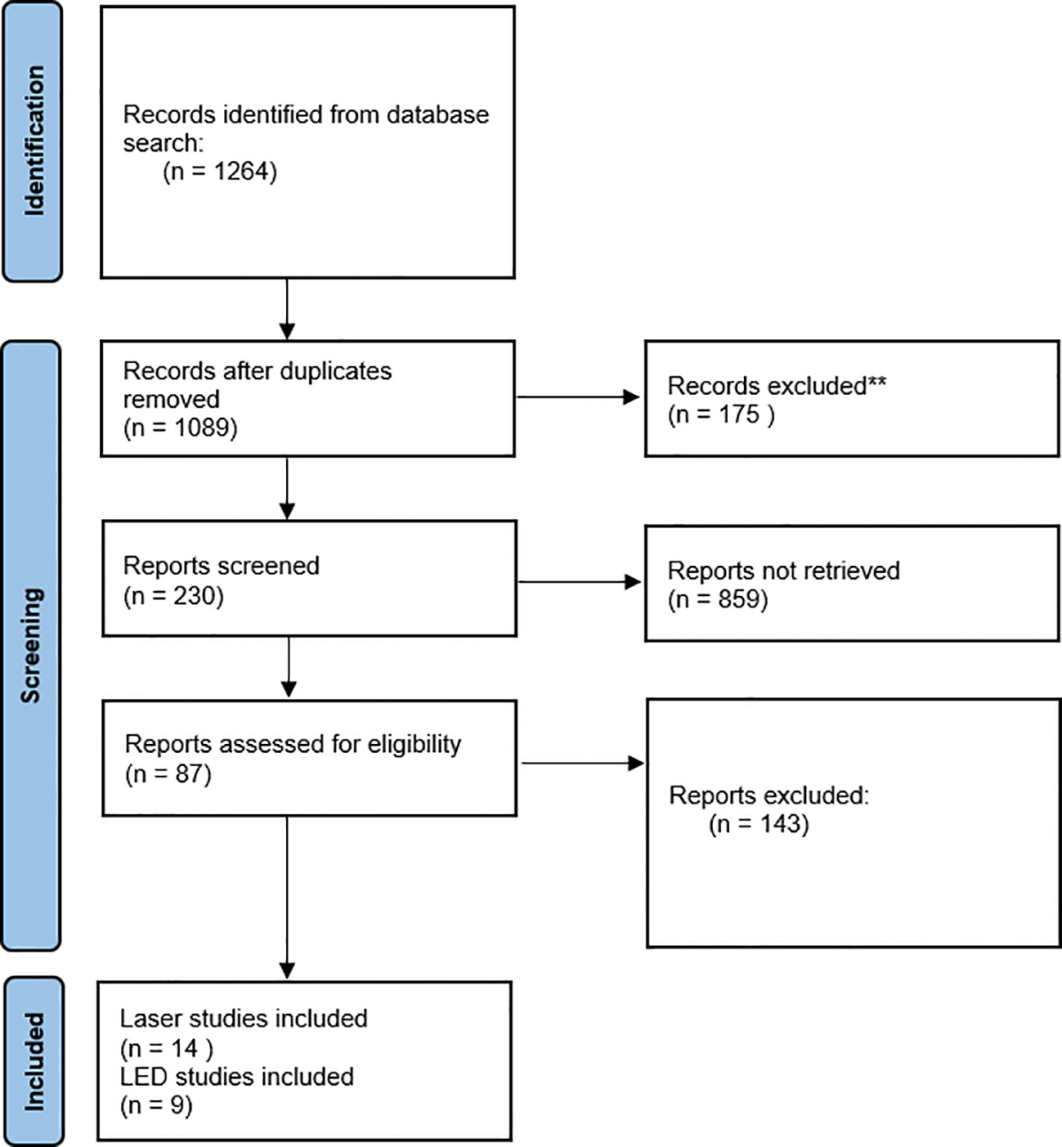
Review search process and winnowing.

The PRISMA flow diagram (**Figure 1**) provides context of the selection workflow. In total, 23 records were included as part of this review, 14 were laser studies and 9 were LED studies.

### Laser PBMT

14 studies examining laser-based PBMT were included. These include *in vitro* and *in vivo* studies and clinical trials.

#### *In vitro* and *in vivo* studies

9 *in vitro* and *in vivo* studies were included.

#### Effects of PBMT on cancer cell viability and proliferation

Several studies investigated the impact of PBMT on cancer cell viability and proliferation, with varying results depending on the cancer type, light parameters, and experimental conditions.

Diniz et al. (14) investigated using PBMT to potentiate the effects of cisplatin chemotherapy on keratinocytes and oral cancer cells. Cells treated with PBMT combined with cisplatin showed increased sensitivity to cisplatin, with enhanced cell death via apoptosis and greater ATP depletion.

(15) found low-dose near-infrared (NIR) laser PBM at 0.3-3 J/cm2 induced significant apoptosis in HeLa cervical cancer cells, with higher rates than non-irradiated controls. Imaging showed nuclear protein reorganization, lipid droplet accumulation, and increased ROS at pro-apoptotic doses, suggesting PBMT triggers cytotoxicity via excessive lipid formation.

Kiro et al. (16) used PBMT to investigate the viability of the treatment on breast and cervical cancer lines. A key focus was targeting therapy resistant cancer stem cells that drive tumor relapse. They found that PBMT decreased cytotoxicity in both breast and cervical cancer stem cells, and increased cell proliferation and viability for both, at all wavelengths.

Kianmehr et al. (17) found low-level 660nm laser irradiation alone didn’t impact viability of normal fibroblasts or melanoma cells. But pre-irradiating melanoma cells with 3 J/cm2 laser before treating with p-coumaric acid selectively reduced their viability via apoptosis, while sparing normal fibroblasts. Suggests laser can sensitize melanoma cells to the anti-cancer effects of p-coumaric acid.

(18) investigated the effects of blue laser (450nm) photobiomodulation on bladder cancer progression. In bladder cancer cell lines T24 and EJ, blue laser irradiation above 4 J/cm2 significantly decreased cell viability and proliferation marker Ki67 in a density-dependent manner, without impacting normal uroepithelial cells until 16 J/cm2.

Gonabadi et al. (19) studied the effects of 650nm and 870nm PBMT on proliferation of HT29 colorectal cancer cells *in vitro* and *in vivo*. The 870nm laser did not significantly impact cultured cell proliferation. However, the 650nm laser promoted proliferation of cultured HT29 cells. Surprisingly, when tested in a mouse xenograft model, the 650nm laser slowed tumor growth compared to controls. This highlights that PBMT’s effects on cancer cell behavior can differ between *in vitro* and *in vivo* models.

The studies collectively demonstrate that PBMT can influence cancer cell viability and proliferation, with effects ranging from increased apoptosis and sensitivity to treatments, to enhanced proliferation depending on the specific parameters used. The variability in results suggests that PBMT’s impact is highly dependent on the type of cancer, laser wavelength, and dosage applied.

#### Combination therapies: PBMT with other agents

Some researchers explored the potential of combining PBMT with other therapeutic agents to enhance anti-cancer effects.

(20) synthesized chitosan-tripolyphosphate nanoparticles (C-TPP NPs) and tested their effects, with and without near-infrared (NIR) laser irradiation, on the viability of colon cancer Caco-2 cells. Characterization showed successful NP synthesis. C-TPP NPs alone decreased Caco-2 cell viability and increased cytotoxicity. NIR laser irradiation alone also reduced cancer cell viability. Microscopy revealed the NPs caused the cancer cells to shine brightly under laser exposure, suggesting potential for cancer detection and treatment using this NP-laser combination approach.

(21) investigated combining low-level 660nm laser irradiation with the natural phenolic compound gallic acid (GA) against breast cancer (MDA-MB-231) and melanoma (A375) cells, as well as normal fibroblasts (HDF) and breast epithelial cells (MCF10A). Pre-treating cancer cells with laser first, then GA reduced viability more than GA followed by laser. The laser + GA combination increased ROS production, apoptosis, and ferroptosis in cancer cells compared to GA alone, while sparing normal cells. This suggests that low-dose laser can sensitize breast and melanoma cancers to the cytotoxic effects of GA via ROS-mediated cell death pathways like apoptosis/ferroptosis, representing a potential therapeutic approach.

These studies highlight the potential of PBMT to enhance the effects of other therapeutic agents by increasing cancer cell susceptibility and promoting cell death. The combination of PBMT with other treatments appears to offer a synergistic effect that could be leveraged for more effective cancer therapies.

#### PBMT effects on cancer treatment and progression

Additionally, some studies focused on how PBMT might interact with other cancer treatments or influence cancer progression.

Barasch et al. (22) used an orthotopic mouse model of oral squamous cell carcinoma to test if PBMT protects tumors from radiation therapy (RT). Mice with tumors received PBM alone, RT alone, or PBMT + RT at various parameters. RT significantly improved survival and reduced tumor volume vs control and PBM-only groups. Crucially, no differences were seen between RT alone vs PBMT + RT groups, indicating PBM did not protect tumors from RT’s anti-cancer effects at the doses tested.

(18) in addition to their findings on cell viability, discovered that blue laser at 4-8 J/cm2 reduced bladder cancer cell migration, invasion, and epithelial-mesenchymal transition (EMT) by downregulating MMP-2/9, Snail, N-cadherin and phospho-MEK/ERK while increasing E-cadherin, suggesting inhibition of cancer progression via suppression of the MAPK/MEK/ERK pathway.

The studies suggest that PBMT does not interfere with the effectiveness of traditional cancer treatments like radiation therapy and may even inhibit cancer progression by affecting cellular pathways involved in migration and invasion. These findings indicate that PBMT could be incorporated into existing treatment regimens without diminishing their efficacy.

#### Clinical trials

Genot-Klastersky et al. (11) evaluated the impact of using PBMT with low-level laser therapy on outcomes in head and neck cancer patients receiving radiation therapy with or without chemotherapy. Out of 361 patients analyzed, 222 (62%) received PBM for management of severe oral mucositis while 139 (39%) did not receive PBM. The two groups were balanced for patient characteristics. Multivariate analysis showed no statistically significant differences between PBM and non-PBM groups in overall survival, time to local recurrence, or progression-free survival after adjusting for known prognostic factors. Shown in **Tables 1** and **2**, the results provide evidence that adjunctive use of PBM during radiation/chemoradiation for head/neck cancers had no impact on long-term tumor control or survival outcomes in this patient population. This suggests PBM may only provide supportive care benefits for mucositis without altering anti-tumor efficacy.

**Table 1:** Laser-based PBMT *in vitro* and *in vivo* studies.

Author	Year	Study/Model Type	PBMT Device	Wavelength	Protocol	Study/Conclusion
Diniz et al. (14)	2019	SCC25 Cancer Cells HN12 Cencer cells	Laser	660 nm	Single Application	**“**Cell lineages showed increased sensitivity to cisplatin associated with PBM.” Overall observation suggests that PBM may lead to increased drug cytotoxicity and enhanced cell death.
Levchenko et al. (15)	2019	Human Cervical Cancer Cells (HeLa)	Laser	808 nm	Single Application	Apoptosis was induced by PBMT gradually over time.
Kianmehr et al. (17)	2019	A375 cancer cells SK-MEL-47 cancer cells	Laser	660 nm	Single Application	PBMT alone was not able to kill human melanoma cells and PBMT followed by p-Coumaric acid treatment did not change cell viability.
Kianmehr et al. (17)	2019	A375 cancer cells SK-MEL-47 cancer cells	Laser	660 nm	Single Application	PBMT alone was not able to kill human melanoma cells and PBMT followed by p-Coumaric acid treatment did not change cell viability.
Barasch et al. (22)	2019	Cal-33 cancer cells	Laser	660 nm 850 nm	Daily Application of 660 nm Single application of 660 nm Single Application of 660 nm + 850 nm	No significant difference between control group and PBMT group.
Abuelmakarem et al. (20)	2019	Caco-2 Cancer Cells	Laser	660 nm	Single Application	PBMT decreased cell viability
Kiro et al. (16)	2019	Breast and Cervical Cancer Stem Cells	Laser	636 nm 825 nm 1060 nm	Single Application	PBMT decreased cytotoxicity in both breast and cervical cancer stem cells, and increased cell proliferation and viability for both, at all wavelengths.
Khorsandi et al. (21)	2020	MDA-MB-231 cancer cells A375 Cancer Cells	Laser	660 nm	Single Application	PBMT alone was not able to kill human cancer cells and PBMT followed by gallic acid treatment did not change cell viability.
Xia et al. (18)	2021	T24 and EJ Human Bladder Cell Lines	Laser	450 nm	Single Application	PBMT with blue laser irradiation inhibited cell migration and invasion.
Gonabadi et al. (19)	2023	HT29 Colorectal cancer cells	Laser	650 nm 870 nm	Single Application	Higher wavelengths did not alter cell proliferation, but lower wavelengths did increase cell proliferation. However, lower wavelength did decline the rate of tumor progression

**Table 2:** Laser-based PBMT clinical trials.

Author	Year	Study/Model Type	PBMT Device	Wavelength	Protocol	Study/Conclusion
Genot- Kastersky et al. (11)	2019	Head and Neck Cancer Patients	Laser	630 nm	Three times a week	No significant difference between control group and PBMT group.
Morais et al. (23)	2020	Head and Neck Cancer Patients with oral side effects	Laser	660 nm	Daily Application	PBMT associated with a rigorous preventative oral care program reduced quality of life impact for cancer patients.
Lodewijckx et al. (24)	2020	Patients with chemotherapy induced alopecia	Laser	678 nm	Three times a week	PBMT significantly improved quality of life for cancer patients and accelerated hair regrowth after chemotherapy.
Kuhn-Dall’Magroet al. (25)	2022	Head and Neck Cancer Patients	Laser	660 nm 810 nm 660 + 810 nm	Daily Application	PBMT was effective in treating oral mucositis in cancer patients.
de Carvalho E Silva et al. (26)	2023	Cancer Patients with xerostomia and oral mucositis	Laser	660 nm	Weekly application	PBMT improved Quality of Life for cancer patients and lower cases of xerostomia and oral mucositis were seen in PBMT groups compared to control groups.

(23) evaluated the effects of a preventive oral care program (POCP) combined with PBMT in 61 head/neck cancer patients undergoing radiochemotherapy. The POCP included oral hygiene, infection control, fluoride, hydration, and denture removal, plus daily PBMT. At baseline, no patients had oral mucositis (OM). Only 45.9% developed OM by the 7th radiotherapy (RT) session, with few severe cases. OM symptoms like pain, dysphagia, dysgeusia progressively increased until the 14th RT session then plateaued. Quality of life was similarly impacted. Only 3 patients (5%) required RT interruption due to OM for ≤10 days. The POCP was effective for plaque control and gingival inflammation. Overall/disease-free survival rates were 77%/73.8%. The findings suggest this POCP+PBMT approach provided satisfactory control of oral complications, limited quality of life impacts, and minimized RT interruptions in head/neck cancer patients.

Lodewijckx et al. (24) conducted a randomized placebo-controlled pilot trial evaluating photobiomodulation (PBM) for preventing chemotherapy-induced peripheral neuropathy (CIPN) in 32 breast cancer patients. The PBM group received twice-weekly PBM during chemotherapy, while controls got placebo treatments. Compared to controls, the PBM group had significantly better quality of life scores, less worsening of sensory neuropathy symptoms, and faster recovery of functional deficits like 6-minute walk distance and pain levels after chemotherapy completion. The promising results suggest PBM may help prevent CIPN development and associated impairments in breast cancer patients undergoing chemotherapy.

25 evaluated different low-level laser therapy (LLLT) protocols for managing radiation-induced oral mucositis (OM) in head/neck cancer patients. 80 patients receiving radiotherapy +/- chemotherapy were randomized into 3 groups: 660nm (red), 810nm (infrared), or combined 660/810nm LLLT for 42 days. The combined 660/810nm group had significantly lower OM scores compared to single wavelength groups, though pain scores were similar across groups. The results suggest multi-wavelength LLLT provides better control of OM lesions than single wavelengths, potentially improving quality of life during cancer treatment in the oral cavity.

de Carvalho E Silva et al. (26) evaluated PBMT for managing xerostomia and oral mucositis (OM) in 53 head/neck cancer patients undergoing radiotherapy. Patients were randomized to PBM-T or sham groups, both receiving artificial saliva. The PBM-T group showed significantly improved quality of life (QoL) scores and less severe xerostomia symptoms compared to sham controls whose QoL worsened over time. Higher grades of OM were observed in the sham group versus the PBM-T group. No significant differences were found in dental caries (DMFT) or periodontal indices between groups. Overall, the results suggest PBM-T can help mitigate radiotherapy-induced xerostomia and OM, improving QoL outcomes in head/neck cancer patients compared to artificial saliva alone.

These clinical trials consistently demonstrate that PBMT, especially when combined with preventive oral care or using multi-wavelength approaches, can effectively reduce the incidence and severity of oral mucositis and xerostomia in head and neck cancer patients undergoing radiotherapy or chemoradiotherapy. This leads to improved quality of life and fewer treatment interruptions.

### LED PBMT

As shown in **Table 3** and **Table 4**, 9 studies examining laser-based PBMT were included. These include *in vitro* and *in vivo* studies and clinical trials.

**Table 3:** LED-based PBMT *in vitro* and *in vivo* studies.

Author	Year	Study/Model Type	PBMT Device	Wavelength	Protocol	Study Conclusions
Chen et al. (27)	2019	B16F10 melanoma cells	LED	Two continuous blue waves (418 nm and 457 nm), one continuous red wave (630 nm)	Single application	PBMT inhibited the growth of melanoma cancer cells
Takemoto et al. (10)	2019	CAL-27 cells	LED	660 nm	Three applications	PBMT at high doses inhibited the progression and number of oral squamous cancer cell colonies
Matsuo et al. (28)	2019	HSC-3 cells	LED	630 nm	Single Application	HSC-3 cells irradiated with red LED light showed increased migration ability
Shakibaie et al. (4)	2020	MCF-7 Breast Cancer Cells	LED	435 nm 629 nm	Single Application	Lower wavelengths decreased proliferation, but higher wavelengths increased proliferation
Kim et al. (9)	2021	Human pancreatic cancer cells in mouse model	LED	460 nm	Single Application	Blue LED irradiation suppressed pancreatic cancer cell and tumor growth by regulating AKT/mTOR signaling.
Jeon et al. (29)	2020	Human melanoma cells	LED	660 nm	Single Application	PBMT decreased melanoma cell viability
Yoshimoto et al. (30)	2022	HCT-116 colon cancer cells	LEDs	465 nm	Single Application	Blue LED light may have a direct antitumor effect on colon cancer
Kim et al. (31)	2023		LED	450 nm	Single Application	LED irradiation aggravated the defective p53 signaling pathway and induced cell growth arrest in cancer cells. Consequently, cancer cell apoptosis was induced by the increased DNA damage.

**Table 4:** LED-based PBMT clinical trials.

Author	Year	Study/Model Type	PBMT Device	Wavelength	Protocol	Study Conclusions
Guimaraes et al. (30)	2021	Pediatric cancer patients with oral mucositis	LED and Lasers, a comparative study	N.S.	N.S	Incidence of oral mucositis was similar in both groups

#### *In vitro* and *in vivo* studies

##### Effects of PBMT on cancer cell viability and proliferation

(27) investigated how different PBMT parameters like irradiance and dose impacted the inhibitory effects of blue light on B16F10 melanoma cells. They found high irradiance blue light was more effective at inhibiting melanoma cell growth compared to low irradiance at the same total dose levels. The enhanced inhibition with higher irradiance was proposed to be due to increased ROS production disrupting mitochondrial function. Their results suggest that optimizing PBMT irradiance is important for maximizing the anti-melanoma effects of PBMT by modulation ROS generation.

(4) investigated how PBMT at different wavelengths (435 nm blue light vs 629 nm red light) impacted the metabolic activity of MCF7 breast cancer cells. Blue light decreased MCF7 cell viability by 23% compared to controls. It also downregulated the expression of glycolytic genes LDHA and GLS, reducing glucose consumption and lactate production. In contrast, red light (629nm) upregulated LDHA/GLS, increasing glucose uptake/lactate secretion. HPLC analysis showed that blue light decreased while red light increased glutamine consumption by MCF7 cells.

(9) found that blue light LED irradiation (460nm) suppressed proliferation and induced apoptosis in pancreatic cancer cells. This was mediated by downregulating mutant p53, Bcl-2, AKT2, phospho-AKT, and mTOR - key proteins involved in survival signaling. Blue LED also increased cleavage of apoptosis executioners PARP and caspase-3. In a pancreatic cancer xenograft model, blue LED inhibited tumor growth associated with reduced AKT2 levels.

(31) proposed using low-energy white light LED irradiation as a moderate approach to selectively inhibit cancer cell proliferation without affecting normal cells. *In vitro* experiments showed LED exposure aggravated defective p53 signaling and induced growth arrest/apoptosis in HeLa cervical cancer cells by increasing DNA damage. LED irradiation also suppressed the MAPK pathway to block cancer cell proliferation. Importantly, in a cancer xenograft mouse model, LED light inhibited tumor growth, associated with modulation of p53 and MAPK pathways.

These studies demonstrate that blue light, particularly at higher irradiances, generally inhibits cancer cell viability and alters metabolic activity by affecting mitochondrial and glycolytic pathways. In contrast, red light tends to enhance metabolic activity.

### Combination therapies: PBMT with other agents

(29) presents a parallel-stacked OLED design that achieves high power output at low driving voltages for a novel wearable device. The work reported high singlet oxygen generation was 3.8x higher than a reference OLED, confirming PDT potential - reducing melanoma cell viability by 24% after a 0.5-hour irradiation, when doing an *in vitro* study.

These studies suggest that PBMT, particularly blue light, holds promise for photodynamic therapy and tumor growth suppression, with effects potentially mediated by increased reactive oxygen species and modulation of autophagy and survival pathways. This highlights LED-based blue light PBMT’s potential as a non-invasive therapeutic strategy in oncology.

### PBMT effects on cancer treatment and progression

(10) investigated using high-dose LED PBMT to inhibit progression of oral potentially malignant disorders tom invasive carcinoma. *In vitro* co-culture models were used with oral squamous cell carcinoma and fibroblast stroma. High-dose PBMT inhibited expansion of carcinoma *in situ* colonies and reduced total colony number after 72 hours compared to untreated controls. While the PBM treatment impacted carcinoma cell viability and induced apoptosis, it had less effect on the surrounding fibroblast stroma cells.

(28) found that irradiating the oral squamous cell carcinoma (OSCC) cell line HSC-3 with red LED light (630nm) increased their migration ability *in vitro*. Interestingly, this was associated with induced expression of the cytokine interleukin-6 (IL-6), which promotes cancer cell migration. Their results suggest red LED photobiomodulation may have the undesirable effect of enhancing the invasive potential of OSCC cells, potentially via an IL-6 mediated mechanism. This raises safety concerns about using red light therapy for OSCC which warrants further investigation.

(30) investigates the effects of 465nm blue LED light irradiation on human colon cancer HCT-116 cells and the tumor microenvironment in a mouse xenograft model. Blue LED light suppressed tumor growth *in vivo*, increased expression of the light-sensitive opsin 3 protein, and induced autophagy gene expression in tumors. Importantly, blue LED reduced expression of cancer-associated fibroblast (CAF) activation markers like α-SMA and IL-6 in the tumor stroma. *In vitro*, blue LED irradiation of CAFs prevented their ability to promote colon cancer cell migration, invasion and PD-L1 upregulation.

Overall, while blue light appears to suppress cancer cell migration and invasion, red light may enhance these processes, potentially via cytokine-mediated mechanisms. These findings highlight the importance of selecting appropriate wavelengths to avoid undesirable effects on cancer invasiveness.

### Clinical trials

(32) conducted a randomized study compared the efficacy of low-level laser therapy (LLLT) versus light-emitting diode therapy (LEDT) for preventing and treating oral mucositis in pediatric leukemia patients receiving high-dose methotrexate chemotherapy. 80 patients were divided into LLLT and LEDT groups, receiving the same energy/radiant exposure parameters. The incidence of developing oral mucositis was similar between LLLT (10%) and LEDT (12.5%) groups. Both required the same number of days for mucositis and pain resolution based on WHO and VAS scores. No significant differences were found between LLLT and LEDT in preventing/treating oral mucositis or associated pain levels. The findings suggest LEDT can be an effective alternative to LLLT photobiomodulation for managing this complication in pediatric cancer patients undergoing aggressive chemotherapy regimens.

The clinical trials reviewed in this study predominantly focused on photobiomodulation therapy (PBMT) for managing side effects of cancer treatments, particularly in head and neck cancer patients. These trials consistently demonstrated PBMT’s efficacy in reducing the incidence and severity of oral mucositis and xerostomia, leading to improved quality of life and fewer treatment interruptions. Importantly, studies investigating long-term outcomes found that PBMT did not negatively impact cancer treatment efficacy or survival rates. This highlights a significant opportunity in clinical research specifically examining LED-based PBMT in oncology settings, despite the growing body of preclinical evidence supporting its potential benefits.

## Discussion

### Findings

Both laser- and LED-based PBMT demonstrated therapeutic potential. The laser-based PBMT approaches varied between *in vitro* studies, vivo studies, and clinical trials. The LED-based PBMT demonstrated results that varied with light wavelength. Findings suggest the potential that utilizing PBMT with multiple wavelengths may have complementary effects (25). Outside of direct cancer treatment, the ability of PBMT to treat secondary effects of chemotherapy was well documented (23, 25).

### Laser PBMT

The *in vitro* and *in vivo* studies demonstrated laser PBMT’s potential to selectively induce apoptosis and cytotoxicity in various cancer cells, including enhancing sensitivity to chemotherapeutics like cisplatin (14) and natural compounds like p-coumaric acid (17) and gallic acid (21). However, Kiro et al. (16) found PBMT decreased cytotoxicity in breast and cervical cancer stem cells. Notably, Gonabadi et al. (19) highlighted divergent effects of 650nm laser irradiation on colorectal cancer cell proliferation between *in vitro* (promoted proliferation) and *in vivo* xenograft models (slowed tumor growth), underscoring the importance of validating findings across multiple experimental settings.

Clinical trials provided evidence that adjunctive laser PBMT during radiation/chemoradiation for head/neck cancers did not impact long-term tumor control or survival (11). Instead, PBMT demonstrated benefits in mitigating treatment side effects like oral mucositis (23, 25), xerostomia (26), and chemotherapy-induced peripheral neuropathy in breast cancer patients (24). Multi-wavelength protocols provided better mucositis control than single wavelengths (25).

### LED PBMT

The studies by Chen et al. (27) and Kim et al. (2021) demonstrated the potential of blue LED light in inhibiting melanoma and pancreatic cancer cell proliferation and inducing apoptosis, potentially mediated through ROS generation and survival signaling modulation. However, Matsuo et al. (28) raised concerns about red LED enhancing oral squamous cell carcinoma migration and invasiveness via an IL-6 mediated mechanism. Yoshimoto et al. (30) showed blue LED irradiation suppressed colon cancer tumor growth *in vivo* and reduced cancer-associated fibroblast activation markers in the tumor microenvironment. The clinical trial by Guimaraes et al. (32) found no significant differences between low-level laser therapy (LLLT) and LED therapy in preventing/treating oral mucositis in pediatric leukemia patients, suggesting LED-PBMT may be an effective alternative to laser approaches.

Interestingly, Jeon et al. (29) presented a novel parallel-stacked OLED (PAOLED) design that achieved high singlet oxygen generation, around 3.8 times higher than a reference OLED. This high singlet oxygen output reduced melanoma cell viability by 24% after just 0.5 hours of irradiation *in vitro*, highlighting the potential of OLEDs as wearable photodynamic therapy devices for cancer treatment.

## Findings

This review of recent literature highlights the therapeutic benefits of PBMT in oncological clinical settings. PBMT is already being used in clinical applications for managing side effects induced by cancer treatment. Nonetheless, there is a clear need for further investigation, particularly regarding the clinical use of LEDs as an alternative irradiation source to lasers.

In clinical studies, laser-based PBMT has been studied more extensively. In literature comparing the clinical viability of LED PBMT (Guimaraes, 2020), it has been shown that LED irradiation shows comparable results to laser irradiation. LED irradiation also offers several practical advantages, including lower costs, increased safety, and the potential for wearable or portable devices (29). While there are many *in vitro* studies of blue and red LED irradiation, there seems to be a limited number of studies evaluating LED PBMT in clinical trials. Additionally, there have been advances in LED technology in terms of wearable technology.

## Limitations

The current review had several inherent limitations that should be acknowledged. Firstly, the scope of the investigation was relatively narrow, and the findings may be susceptible to bias due to the small sample size of studies included. The incorporation of both *in vitro* and *in vivo* studies posed challenges in appropriately assessing and accounting for potential biases across different experimental settings and methodologies. Moreover, there was a notable disparity in the number of clinical trials evaluating LED irradiation compared to those investigating laser-based PBMT. This imbalance in representation could potentially skew the overall conclusions and limit the generalizability of the findings to different light modalities.

Additionally, while this review aimed to assess the overall efficiency of PBMT in oncological treatments, it is important to note that the effects of PBMT are influenced by multiple parameters beyond just wavelength. The review’s initial focus on wavelength may have led to an underrepresentation of other crucial factors such as intensity, duration of treatment, among other parameters. Nonetheless, the heterogeneity in reporting standards across studies, particularly regarding the detailed specifications of PBMT parameters, posed challenges in making direct comparisons across studies among multiple PBMT parameters.

## Future work

Further steps are necessary to comprehensively evaluate the viability and efficacy of PBMT in cancer treatment, several key aspects warrant further investigation. Firstly, a more refined scope and stringent eligibility criteria are necessary to delineate the precise effects of LED versus laser irradiation. The current review has unveiled instances where PBMT outcomes diverge between *in vivo* and *in vitro* settings for the two light modalities. It is crucial to note that lasers emit coherent light, while LEDs produce incoherent light. Although some studies have reported similar results with lasers and LEDs in the context of PBMT, the intrinsic differences in light parameters between these modalities could potentially influence tumor responses.

Additionally, future research should explore the differential effects of pulse versus continuous wave lasers/LEDs on cancer sites. The trials presented herein primarily focused on continuous light waves. However, the pulsing characteristics of light sources may play a pivotal role in modulating cellular responses and therapeutic outcomes.

Furthermore, a comprehensive examination of the impact of various light parameters, such as wavelength, fluence, irradiance, and treatment regimen, on specific cancer types and stages is warranted. This approach would facilitate the optimization of PBMT protocols tailored to individual clinical scenarios, potentially enhancing therapeutic efficacy and minimizing adverse effects. The combination of optical wavelengths for PBMT may offer therapy with complementary mechanisms.

Overall, as the field progresses towards more LED-based clinical trials, there should be a particular focus on investigating the effects of blue LED irradiation in oncological settings. This emphasis is warranted given the promising results observed in preclinical studies. However, before advancing to large-scale clinical trials, it is crucial to conduct a more comprehensive examination of the impact of various PBMT parameters. This should include a thorough investigation of wavelength, fluence, dose, duration, and other treatment regimen factors in both *in vitro* and *in vivo* studies.

## Conclusions

The collective evidence highlights PBMT’s promising potential as an adjunctive therapy in cancer management using both lasers and LEDs. Preclinical studies demonstrated laser PBMT’s ability to induce apoptosis and cytotoxicity in various cancer cells while enhancing sensitivity to chemotherapeutics. However, divergent effects were observed between *in vitro* and *in vivo* models for certain laser wavelengths. LED PBMT studies showed blue light inhibited melanoma and pancreatic cancer growth, potentially via ROS generation, while raising concerns about red light enhancing oral cancer invasiveness. Clinically, laser and LED PBMT did not impact long-term tumor control or survival in head/neck cancer patients undergoing radiation/chemoradiation. Instead, both modalities mitigated treatment side effects like oral mucositis, xerostomia and neuropathy, improving quality of life. While promising, outcomes varied based on light parameters, cancer type and experimental setting. Further well-designed studies optimizing PBMT protocols are needed to establish long-term safety and efficacy across clinical scenarios. Additionally, LED modalities were underreported in clinical settings, which provides an emerging field of research, especially since the development of LED based wearable medical devices.
